# Effect of Abrasive Machining on the Electrical Properties Cu_86_Mn_12_Ni_2_ Alloy Shunts

**DOI:** 10.3390/ma10080876

**Published:** 2017-07-29

**Authors:** Siti Nabilah Misti, Martin Birkett, Roger Penlington, David Bell

**Affiliations:** 1 Department of Mechanical and Construction Engineering, Northumbria University, Newcastle upon Tyne NE1 8ST, UK; martin.birkett@northumbria.ac.uk (M.B.); r.penlington@northumbria.ac.uk (R.P.); 2 Vice Chancellors Office, Teesside University, Middlesbrough TS1 3BA, UK; david.bell@tees.ac.uk

**Keywords:** abrasive machining, Manganin alloy, shunt resistor

## Abstract

This paper studies the effect of abrasive trimming on the electrical properties of Cu_86_Mn_12_Ni_2_ Manganin alloy shunt resistors. A precision abrasive trimming system for fine tuning the resistance tolerance of high current Manganin shunt resistors is proposed. The system is shown to be capable of reducing the resistance tolerance of 100 μΩ shunts from their standard value of ±5% to <±1% by removing controlled amounts of Manganin material using a square cut trim geometry. The temperature coefficient of resistance (TCR), high current, and high temperature performance of the trimmed shunts was compared to that of untrimmed parts to determine if trimming had any detrimental effect on these key electrical performance parameters of the device. It was shown that the TCR value was reduced following trimming with typical results of +106 ppm/°C and +93 ppm/°C for untrimmed and trimmed parts respectively. When subjected to a high current of 200 A the trimmed parts showed a slight increase in temperature rise to 203 °C, as compared to 194 °C for the untrimmed parts, but both had significant temporary increases in resistance of up to 1.3 μΩ. The results for resistance change following high temperature storage at 200 °C for 168 h were also significant for both untrimmed and trimmed parts with shifts of 1.85% and 2.29% respectively and these results were related to surface oxidation of the Manganin alloy which was accelerated for the freshly exposed surfaces of the trimmed part.

## 1. Introduction

Shunt resistors have been used as ammeters to measure the flow of electrical current for several decades and offer the advantages of lower cost, low power loss, high stability, and precision of electric resistance across a wide temperature range. This is significant when compared to other current sensing methods such as current transformers, Hall sensors, and Rogowski coils [1]. Shunt resistors are typically manufactured from a Manganin (Cu_86_Mn_12_Ni_2_ wt %) alloy element which is an electron beam welded to two low resistivity copper terminations to permit accurate electrical measurement. Manganin is a commercially available alloy of resistivity 48.2 × 10^−8^ Ωm [2] and low Temperature Coefficient of Resistance (TCR) of ±15 ppm/°C [3], offering low and stable resistance across a wide temperature range. It also possesses an extremely low thermal electromotive force (EMF) of 0.1 μV/K at 20 °C and excellent long-term stability of electrical resistance. 

One recent important use of Manganin shunts is in single phase smart energy meters to measure the flow of electrical current. Due to its relatively low cost, the shunt resistor is the preferred current sensing method in this application where it must maintain a stable and repeatable resistance value over a wide current range of 0 to 100 A, across an operating temperature of −25 to +55 °C and relative air humidity of 30 to 100% [4]. In order to minimize overall power consumption of the smart energy meter, the room temperature resistance value of the shunt must be as low as possible and typically in the range 100 μΩ to 10 mΩ [5]. Although this low resistance requirement reduces energy usage, it also causes two significant issues. Firstly, the voltage drop that must be sensed across the shunt is substantially reduced, this in turn leads to a requirement to incorporate more accurate measuring equipment into the energy meter. The second issue, which is the focus of this current work, is that it is inherently difficult to manufacture shunt resistors in this low resistance range to the required precision and at a reasonable cost. Typical resistance accuracy of commercially available shunts suitable in this application is 100 μΩ to a tolerance of ±5% [6]. When used to measure current flow in the smart energy meter, this tolerance can result in power usage being either over or under calculated by up to 5%. The current method used to reduce this inaccuracy is to calibrate the overall performance of the assembled meter. This is not always accurate across the full operating conditions and involves the addition of compensation software which increases the overall cost of the meter. A more efficient way to improve the accuracy of the smart energy meter would be to reduce the resistance tolerance of the Manganin shunt resistor itself to less than ±5%.

There are a number of well-established methods of improving the resistance accuracy of thin and thick film discrete resistors by removing resistive material and adjusting the geometry of the element to increase its resistance. The most popular of these techniques are laser trimming, abrasive trimming with a wheel, or abrasive particles and machining [7]. In the majority of processes, the material is removed in single or multiple lines cut perpendicular to the flow of current to give high rates of resistance change or in parallel with the current flow to give slower rates of change [8]. Although these methods have yielded excellent results when adjusting the resistance tolerance of thin and thick film resistors, there has been limited application in the area of bulk metal alloy shunts.

Recent work by the authors has highlighted the potential of a new trimming approach using different geometry cuts to adjust the resistance tolerance of shunt resistors [9]. This current study will investigate the effect of abrasive machining using a square cut geometry on the principal electrical properties of 100 μΩ Manganin alloy shunts.

## 2. Experimental Procedures

### 2.1. Materials

**Figure 1:**
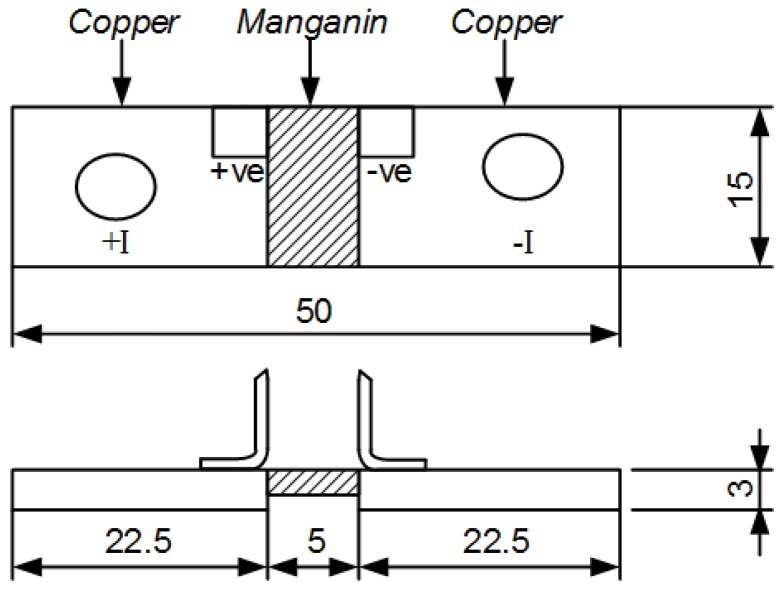
Manganin Alloy Shunt Construction (dimensions in mm).

All samples used in this investigation were constructed from a 15 × 5 × 3 mm thick Manganin alloy element which was electron beam welded to two 22.5 × 15 × 3 mm thick low resistance copper terminations. This produces a 100 μΩ ± 5%, 3 W rated shunt resistor as shown in Figure 1. The resistance values of a batch of 50 shunts were measured to find a sample of 20 parts in the range 95–99 μΩ which were suitable for abrasive trimming to a target value of 100 μΩ.

### 2.2. Abrasive Trimming System

**Figure 2:**
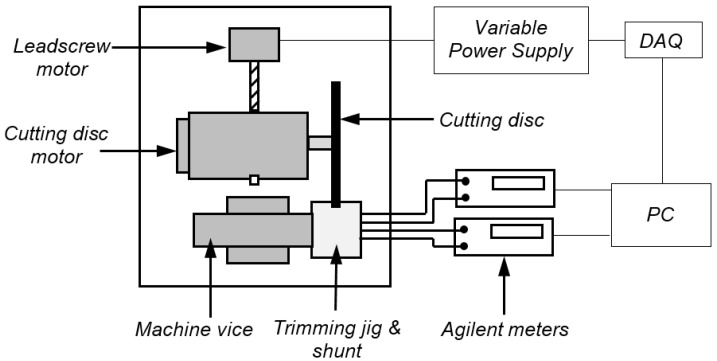
Schematic of the concurrent trimming system.

The experimental setup for the concurrent trimming process used in this study is shown in Figure 2. The shunt samples were trimmed using a Buehler Isomet 5000 linear precision saw fitted with a 178 mm diameter, 0.8 mm thick, rubber bonded silicon carbide (R/SiC) AcuThin cutting disc. The samples were mounted in a PTFE insulated trimming jig which was secured in the machine vice and the cutting disc was rotated at a speed of 3000 rpm and fed into the side of the shunt at a feed rate of 1.2 mm/min. The feed was managed via a leadscrew and drive motor which was driven by a variable power supply unit, and controlled using a program developed in LabVIEW software on a PC.

**Figure 3:**
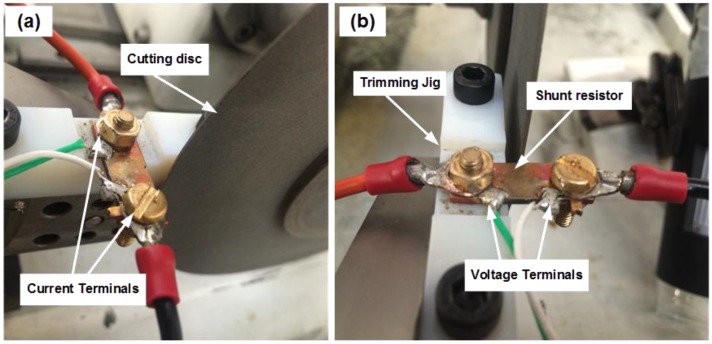
**(a)** Side and **(b)** Front views of the concurrent trimming system.

The resistance value of the shunt was continuously measured during trimming using the combination of an Agilent B2900A source meter and 34420A nanovolt meter. All resistance measurements were performed using the four-wire Kelvin method; a fixed current of 1 A was supplied to the current (I) terminals of the shunt by the B2900A source meter, whilst the voltage drop across the pre-soldered voltage (V) terminals was continuously monitored using the 34420A nanovolt meter. These current and voltage measurements were simultaneously read by the PC and LabVIEW software using a Keysight USB/GPIB interface and were used to calculate the resistance value of the shunt being trimmed. Once this calculated resistance value reached the target value of 100 μΩ, the PC sent a signal to the variable power supply to reverse the voltage supply to the leadscrew motor and retract the cutting disc from the shunt. Figure 3 shows side and front views of the shunt during the concurrent trimming process.

### 2.3. Characterization

Following trimming, the resistance accuracy and distribution of the samples were determined before the shunts were subjected to a series of further tests to establish if trimming causes any significant change to the electrical properties of the components when compared to untrimmed parts. All resistance measurements were achieved using the four-wire Kelvin method and the Agilent B2900A and 34420A meters described earlier.

TCR measurements were conducted in order to simulate the temperature that the shunt resistors may be subjected to whilst in a smart energy meter. The tests were performed in accordance with MIL STD 202 Method 304, using a Grant LTC1 model GD120-R2 refrigerated bath and re-circulator. Each shunt was submerged in Shell transformer oil type Diala S3 ZX-1G while its resistance value was continuously monitored. The temperature of the oil bath was then increased from −10 to +110 °C in 10 °C increments and allowed to stabilize for 10 min at each measurement point before the resistance value of the shunt was recorded. Equation (1) was then used to express the rate of change in resistance (*ΔR*) value per 1 °C (*TCR*) in the prescribed temperature range (*ΔT*)

(1) $$TCR=\frac{∆R}{R\times ∆T}10^{-6}$$

Next, the shunt resistors were connected to a high current power supply (Glassman LPC 6-220) to determine if trimming the Manganin resistive element has any effect on the temperature rise and thus potential power rating of the shunt when used in the smart energy meter. Prior to testing, a type K thermocouple was resistance spot welded to the rear face of the Manganin element of each shunt to measure its temperature rise. The shunts were then powered up to 4 W (~200 A) at a rate of 10 A per second and allowed to stabilize until the temperature of the Manganin element reached a steady state. Thermal images were then taken using a FLIR T620bx camera. Due to the highly reflective properties of the shunt resistors materials, the samples were all painted with Pyromark 1200 high temperature black coating (ε = 0.97) prior to thermal imaging.

High temperature resistance stability tests were conducted in accordance with BS EN 60115-1 standard. The initial resistance value of the shunts were measured before they were stored in an oven in ambient air for 168 h at an elevated test temperature of 200 °C. During testing, the resistance value of the shunts was measured at time intervals of 24, 48, 72, and 168 h and then compared with the initial resistance values to monitor any change in resistance. 

The morphology and composition of the Manganin shunt samples were investigated using a FEI Quanta 200 scanning electron microscope (SEM) at an accelerating voltage of 20 kV. Chemical composition was acquired using an Oxford Instruments INCA X-ray detector at 8000× magnification for 60 s. Energy dispersive spectroscopy (EDS) analysis measurements were made using a lithium-drifted silicon detector attached to the SEM. Surface roughness measurements were performed in accordance with ISO 4287 standard using an Alicona Infinite Focus microscope at ×10 magnification.

## 3. Results and Discussion

**Figure 4:**
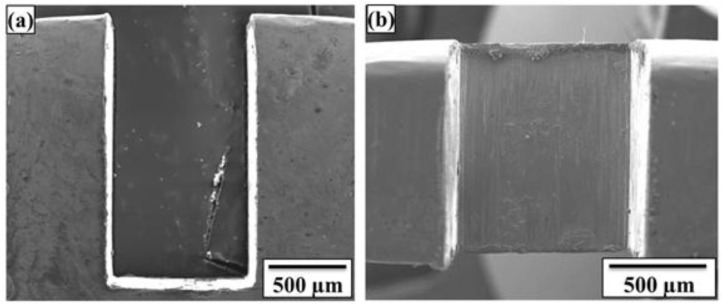
(**a**) Top and (**b**) side views of a shunt resistor trimmed with a square cut.

Top and side views of a typical square cut trim geometry of a Manganin shunt resistor element are shown in Figure 4a,b respectively. The depth of cut for all samples was in the range of 0.5 to 1.2 mm depending on the initial resistance value of the part which equates to around 3 to 8% of the initial Manganin element width of 15 mm. The edges of the trimmed grooves were found to be relatively free from burrs and the surface of the grooves were smooth, having roughness average (Ra) values in the range 100.85 to 133.95 nm.

**Figure 5:**
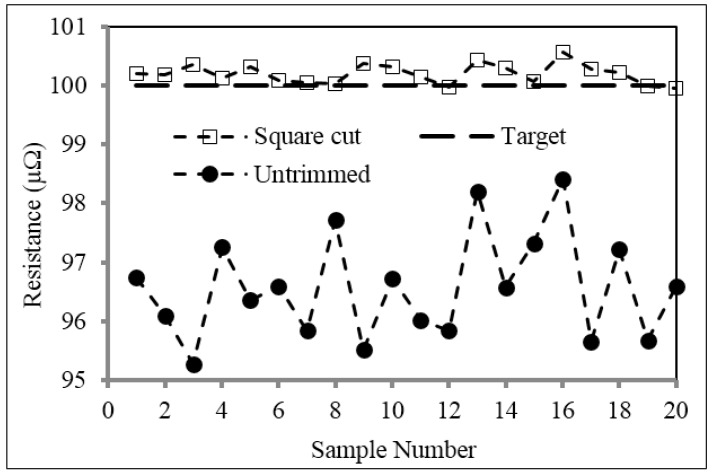
Resistance values of the shunt resistors before (untrimmed) and after trimming with the square cut.

Figure 5 shows the measured resistance value of the 20 samples before (untrimmed) and after trimming with a square cut to a target resistance value of 100 μΩ. It can be clearly seen that the resistance distribution of the sample of shunts after trimming is much smaller than that in the untrimmed condition with values of 95.28 to 98.82 μΩ and 99.96 to 100.56 μΩ, respectively. The resistance values of the trimmed parts are also all well within ±1% of the target resistance value of 100 μΩ, thus highlighting the system’s capability to accurately trim the shunt resistors. 

### 3.1. Temperature Co-Efficient of Resistance (TCR)

**Figure 6:**
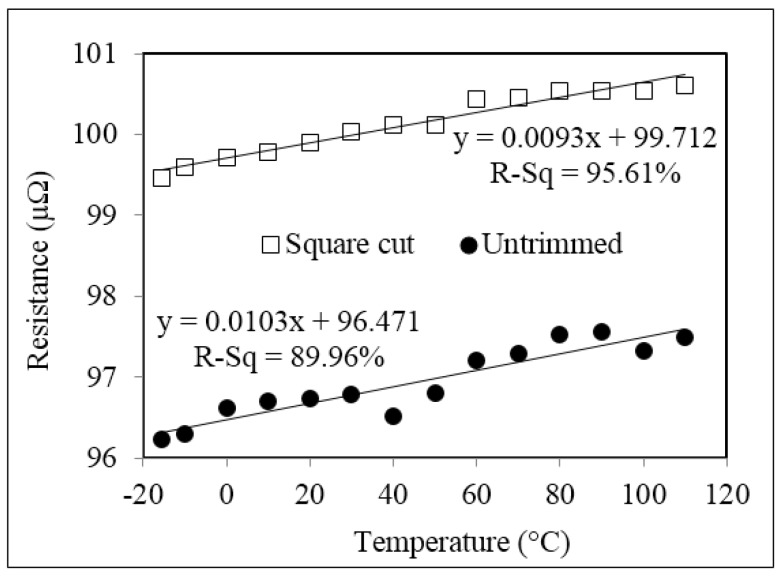
Resistance versus temperature for untrimmed and square cut trimmed shunt resistor samples.

The graph of resistance against temperature in Figure 6 shows a positive correlation for both untrimmed and square cut shunt resistors in the temperature range of −10 to +110 °C. This positive TCR, or decrease in conductivity with an increase in temperature, is typical in metals and alloys and can be related to an increase in energy with temperature which causes the ions to vibrate more frequently and subsequently impede the conduction of electrons [10]. 

It can be further observed that the magnitude of this positive TCR is relatively small across this broad temperature range of −10 to +110 °C, which is a propitious property in the manufacture of precision shunt resistors. The calculated TCR values were consistent across the full temperature range for both the samples of five untrimmed and five square cut trimmed parts, having values in the range of +105 to +108 ppm/°C and +92 to +94 ppm/°C, respectively. The average TCR value for the untrimmed resistors was 106 ppm/°C whereas that for the abrasive trimmed resistors was 93 ppm/°C. These TCR values are higher than those previously reported for pure Manganin of 15 ppm/°C [4], with this increase being related to the series resistance and highly positive TCR (+3930 ppm/°C) contribution of the two small areas of copper termination on either side of the Manganin resistive element [11]. However, the difference in TCR between the untrimmed and trimmed parts, which is the main purpose of this study, is relatively small, at around 13.4 ppm/°C across the full test temperature range of −10 to +110 °C, thus suggesting that trimming has a negligible effect and in fact results in a slight improvement in this key property of the shunts.

### 3.2. High Power Performance

**Figure 7:**
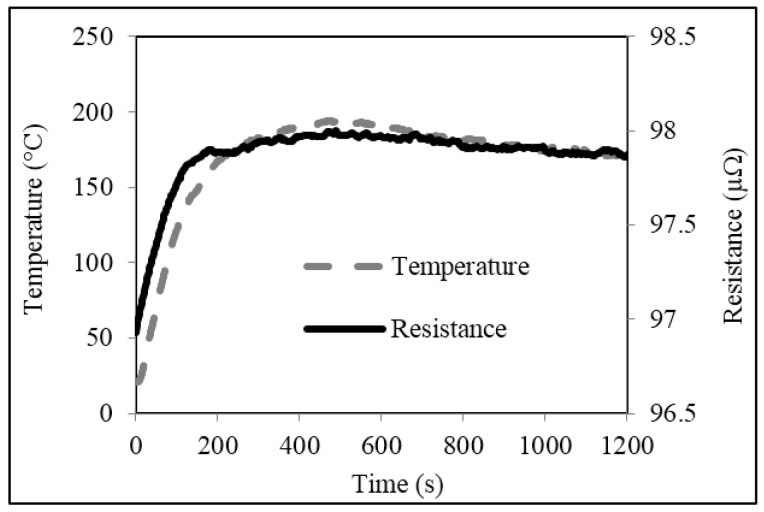
Temperature and resistance versus time plots for the untrimmed shunt resistor.

**Figure 8:**
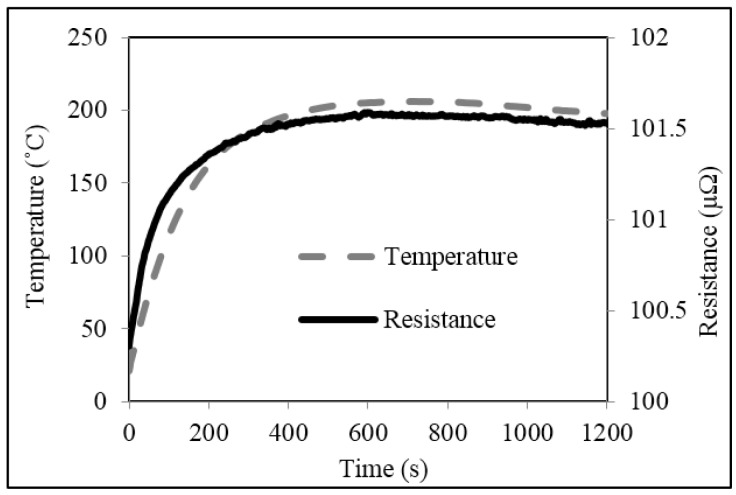
Temperature and resistance versus time plots for the shunt resistor trimmed with the square cut.

Plots of typical temperature increase in untrimmed and square cut trimmed shunt resistors, powered up to 4 W (~200 A), are shown in Figure 7 and Figure 8 respectively. The results for both types of shunt follow a very similar trend. Starting at room temperature (~23 °C) the temperatures of both the untrimmed and trimmed resistor elements increase rapidly in the first 150 s to values around 148 °C and 142 °C respectively. After which, the increases are more gradual before reaching maximum temperatures of 194 °C and 203 °C respectively at around 500 s. The temperature of the two shunts then decreases slightly to around 192 °C for the untrimmed and 198 °C for the trimmed parts and fluctuate around this point until the power supply is switched at a time of 1200 s. After which, both shunts gradually cool back down to room temperature at around 1500 s (not shown on plots).

Plots of resistance versus time for the untrimmed and trimmed shunts, powered up to 4 W, are also shown in Figure 7 and Figure 8 and follow a very similar trend to those described for the temperature rise test. The resistance value of the untrimmed part starts at 96.9 μΩ and rises quickly to 97.8 μΩ within approximately 200 s of the current being supplied. The resistance value of the trimmed part starts at 100.3 μΩ and rises in an almost identical fashion to a value of 101.4 μΩ after 200 s. After this point, the resistance values of both shunts increase more gradually before reaching maximum values of 97.9 μΩ for the untrimmed part and 101.6 μΩ for the trimmed part at around 500 s. After this point, the resistance values of both shunts reduce very slightly to around 97.8 μΩ and 101.5 μΩ respectively and remain there until the power is removed at a time of 1200 s. Finally, the resistance of both shunts was observed to gradually return to their original values as they cool back down to room temperature at around 1500 s (not shown on plots).

**Figure 9:**
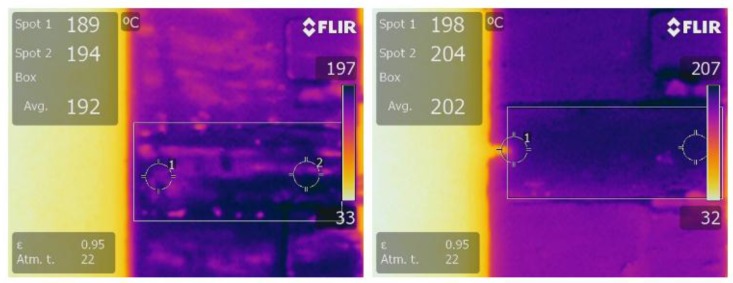
Thermal images of the shunt resistors (**a**) untrimmed and (**b**) trimmed with the square cut.

There are several important observations that can be drawn from the results of Figure 7 and Figure 8. Firstly, the increase in resistance with increase in supplied current is significant for both untrimmed and trimmed parts, having a maximum value of around 1 to 1.3 μΩ (+1 to 1.3%). These increases can be quite clearly related to the inherent TCR of the Manganin material discussed in Section 3.1; multiplying the measured TCR of approximately +100 ppm/°C by the maximum temperature rise of 180 °C gives an increase in resistance of 1.8%. Although this change can be deemed non-permanent, as the resistance of the shunts return to their original values once the power is removed, this temporary increase in resistance could in turn lead to errors when measuring current flow in a smart energy meter. The second observation to be made is that trimming the shunt appears to give a slight increase in the peak temperature rise of the resistor element. This is further supported by the thermal images of the untrimmed and trimmed parts in Figure 9, taken after 500 s when the shunts had reached their maximum temperature.

The temperature was measured at two spots on either side of the centre of the resistor element for both the untrimmed and square cut trimmed parts. For the untrimmed part in Figure 9a, at spot 1, the temperature was 189 °C and at spot 2, it was 194 °C. On average, the temperature was 192 °C. In contrast to the untrimmed part, for the square trimmed part in Figure 9b, at spot 1, which was located next to the end of the trim plunge, the temperature was 198 °C and at spot 2, it was 204 °C. On average, the temperature was 202 °C, which is 10 °C higher than that for the untrimmed shunt. This increase in temperature for the trimmed shunt is comparable with that reported in Figure 7 and Figure 8 and may be attributed to the square shaped plunge cut which impedes the flow of current through the resistive material and causes local current crowding in the central region of the element [12]. This inferior performance could be a cause for concern as increased operating temperature is known to lead to accelerated ageing and premature failure of resistive devices [13]. This issue is discussed further in the following section.

### 3.3. High Temperature Performance

**Figure 10:**
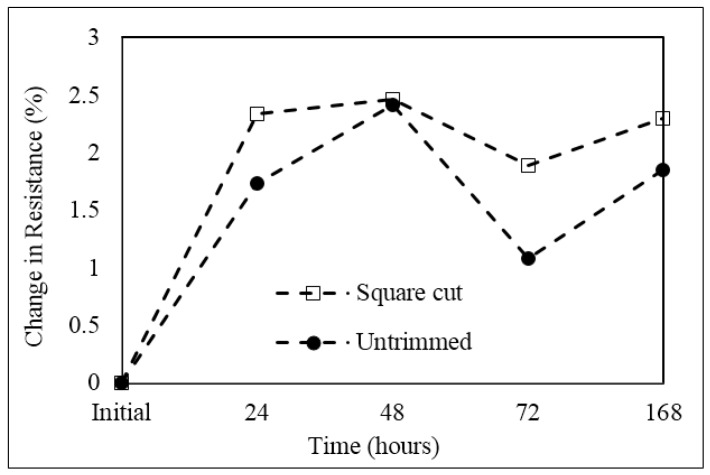
Average change in resistance for untrimmed and square cut trimmed shunt resistor samples during storage at 200 °C for 168 h.

Results of average change in resistance of both the samples of five untrimmed and five square cut trimmed shunt samples following storage for 168 h at 200 °C in air are shown in Figure 10 with interim results reported at time intervals of 24, 48, and 72 h. The graph shows that the average resistance values of both the untrimmed and square cut trimmed shunt samples increase significantly in the first 24 h of the test, with changes of 1.7% and 2.3% respectively. This rate of change reduces significantly in the following 24 h of testing and both samples reach a maximum value of change in resistance of 2.5% after 48 h of storage. After this point, the resistance value of both shunts decreases following 72 h of storage before increasing again to give final changes in resistance values after 168 h of 1.85% and 2.29% for the untrimmed and square cut trimmed shunt samples respectively.

This initial increase and then subsequent decrease in resistance can be related to two competing mechanisms. Initial oxidation of the surface of the Manganin element, resulting in the formation of a high resistance oxide layer, which in turn increases the overall resistance of the material, and annealing out of impurities and grain boundary reduction both of which lead to a reduction in resistance of the material [14]. The higher rate of change of resistance in the first 24 h for the trimmed shunt can be related to an increased level of surface oxidation of the freshly exposed Manganin surrounding the trim cut. This theory is supported by the EDS chemical analysis of oxygen on the shunts’ surfaces, a summary of the results of which are reported in Table 1.

The results show that the untrimmed and square cut trimmed samples have similar average levels of oxygen concentration prior to testing at 3.43 wt % and 5.53 wt % respectively. However, these levels increase significantly for both samples after storage at 200 °C for 168 h with values of 11.92 wt % and 14.71 wt % respectively.

**Figure 11:**
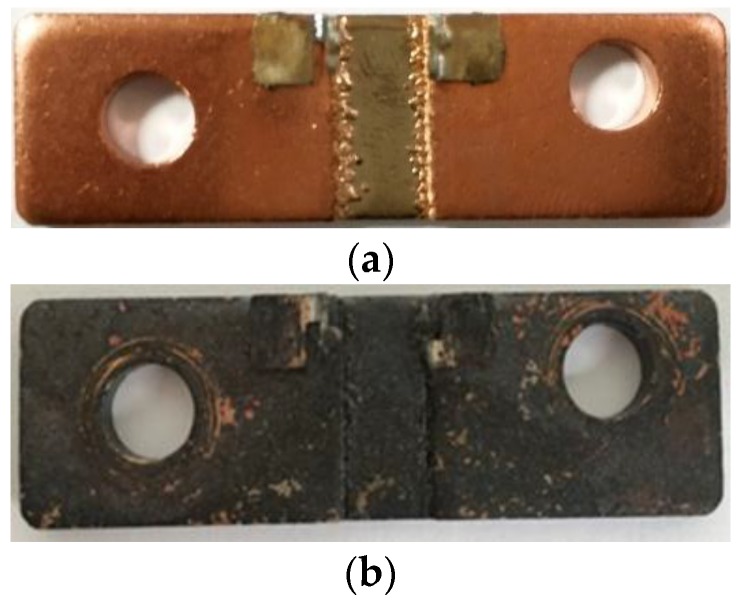
Images of shunt resistor (**a**) before and (**b**) after the high temperature stability test.

These results are further supported by the images of the untrimmed parts before and after testing shown in Figure 11, which clearly show a discoloration and formation of a surface oxide after high temperature exposure.

This increase in surface oxidation of the Manganin element at the elevated test temperature leads to an increase in resistance value of the shunts. Although the resistance change of the square cut trimmed sample is slightly higher at 2.29%, the stability performance of the untrimmed part is also unacceptable at 1.85%. This is an area that needs to be addressed before focusing on reducing the additional effect of trimming on this key performance characteristic of the shunts. 

## 4. Conclusions

Work in this paper has demonstrated that abrasive trimming has the ability to reduce the resistance tolerance of 100 μΩ Manganin shunt resistors from typical values of ±5% to less than ±1% whilst having minimal effect on the key electrical properties of the device.

The TCR, high current, and high temperature performance of the square cut trimmed shunts were compared to that of untrimmed shunts. It was found that the average TCR value was slightly reduced following trimming with typical results of +106 ppm/°C and +93 ppm/°C for untrimmed and trimmed shunts respectively. When subjected to a high current of 200 A the trimmed part showed a slight increase in temperature to 203 °C as compared to 194 °C for the untrimmed part but both parts had significant temporary increases in resistance of around 1 to 1.3 μΩ. This result was quite accurately explained using the inherent TCR property of the Manganin material. The results for resistance change following high temperature storage at 200 °C for 168 h were also significant for both untrimmed and trimmed parts with shifts of 1.85 and 2.29% respectively and these results were related to surface oxidation of the shunts which was accelerated for the freshly exposed surfaces of the trimmed part. 

Although this work has demonstrated that trimming does not have any detrimental effect on the electrical performance of the Manganin shunt resistors it has in turn highlighted some significant inherent performance issues with the Manganin resistive alloy material itself. Therefore, future work should focus on improving the high temperature performance of this material as well as further developing the abrasive trimming process itself to produce accurate and reliable shunt resistors for use in high power smart metering applications.

## Figures and Tables

**Table 1:** EDS chemical analysis for oxygen content of untrimmed and square cut trimmed shunt resistor samples before and after the high temperature stability test

Spectrum No.	wt % Oxygen			
	Untrimmed		Square Cut	
	Before	After	Before	After
1	4.42	10.49	6.05	13.95
2	2.01	16.72	3.60	18.53
3	2.29	6.81	3.61	13.87
4	3.08	14.24	5.45	15.39
5	4.60	9.16	6.71	9.83
6	4.17	14.09	7.76	16.69
**Average**	**3.43**	**11.92**	**5.53**	**14.71**
